# iTRAQ-Based Protein Profiling in CUMS Rats Provides Insights into Hippocampal Ribosome Lesion and Ras Protein Changes Underlying Synaptic Plasticity in Depression

**DOI:** 10.1155/2019/7492306

**Published:** 2019-05-02

**Authors:** Jialing Zhang, Zhinan Zhang, Jiping Zhang, Zheng Zhong, Zengyu Yao, Shanshan Qu, Yong Huang

**Affiliations:** ^1^School of Chinese Medicine, The University of Hong Kong, 999077, Hong Kong; ^2^School of Traditional Chinese Medicine, Southern Medical University, Guangzhou, Guangdong Province, 510515, China; ^3^Nanfang Hospital, Southern Medical University, Guangzhou, Guangdong Province, 510515, China

## Abstract

Hippocampal atrophy is one of the key changes in the brain implicated in the biology of depression. However, the precise molecular mechanism remains poorly understood due to a lack of biomarkers. In this research, we used behavioral experiments to evaluate anxiety and anhedonia levels in depressed rats using chronic unpredictable mild stress (CUMS) modeling. We also used isobaric tag for relative and absolute quantitation (iTRAQ) to identify the differentially expressed hippocampal proteins between depressed and normal rats. Bioinformatics analyses were also performed for a better understanding. The results showed that CUMS rats had higher anxiety and anhedonia levels than control rats, along with hippocampal lesions. Through iTRAQ and bioinformatics analyses, we found that ribosome proteins were significantly downregulated and Ras proteins exhibited a mixed change in the hippocampus of depressed rats. These findings suggest that the expression of hippocampal ribosome lesions and Ras proteins is significantly different in depressed rats than in control rats, providing new insights into the neurobiology of depression.

## 1. Introduction

Approximately 50% of suicide victims worldwide suffer from depression or another mood disorder, which makes depression one of the leading causes of disease burden [1, 2]. Efforts have been made to understand the biology of depression. Several theories have been raised regarding the issue. One of the dominant theories is the monoamine hypothesis, which postulates that a deficit of certain neurotransmitters is responsible for depression [3]. The monoamine hypothesis is based on the observation that many antidepressants increase neurotransmitters at synaptic levels [4]. However, the limitation of the monoamine hypothesis is revealed by the 1-2-week therapeutic lag of these antidepressants, such as selective serotonin reuptake inhibitors (SSRIs) [5]. Additionally, researchers have found that serotonin knockout animals do not have typical depressive behaviors [6]. Therefore, the monoamine hypothesis might oversimplify the problem.

The inflammation theory has also been raised, stressing the role of inflammation in depression. Various reviews have found that depression patients have high levels of inflammatory cytokines, such as interleukin- (IL-) 1*β*, IL-6, and tumor necrosis factor- (TNF-) *α* [7]. When exposed to stress, the translation of inflammatory cytokines is activated. Overexpressed inflammatory cytokines travel through the blood-brain barrier (BBB) or are released by microglia and influence brain function [8]. Studies have also demonstrated that anti-inflammatory therapies can alleviate the symptoms [9].

Among all these theories, stressor exposure has proven to be the most robust factor associated with the development of depression [10]. In response to stressors, the hypothalamic-pituitary-adrenal (HPA) axis is activated. Long-term exposure causes HPA axis dysfunction and high levels of glucocorticoids, which result in cell loss and compromise neurogenesis in the hippocampus [11]. Therefore, atrophy of the hippocampus is considered one of the main features of the depressed brain, which has been repeatedly observed in humans and rodents [12, 13].

Recent studies have also called attention to the role of disrupted synaptic plasticity (the ability of synapses to strengthen or weaken over time) in depression. Studies have demonstrated that synapse number is significantly reduced in certain crucial brain regions, such as the prefrontal cortex (PFC) and hippocampus [14]. In addition, depressed animals have impaired hippocampal long-term potentiation (LTP), which is a pattern of synaptic activity in which a long-lasting increase in synaptic strength is observed [15].

Proteomic technologies are the ideal techniques for the detection and investigation of biomarker candidates, owing to the high sensitivity and analytical performance that can be achieved and the ability to generate large datasets through the identification of large and ever-increasing numbers of proteins [16–18]. Isobaric tag for relative and absolute quantitation (iTRAQ) is a proteomic approach that can determine the amount of proteins from different sources in a single experiment [19]. This technology has been used to outline the proteomic profiles of cancers [20]. Currently, a number of biomarkers for bladder cancer have been detected in urine and tissue using this technique [21].

In this study, we identified differentially expressed proteins in the depressed and normal hippocampus using iTRAQ. Regarding the abundance of the proteins and their biological information, bioinformatics analyses were performed to identify possible proteins underlying the biology of depression.

## 2. Methods

### 2.1. Animals

A total of 25 Wistar rats (males; weight 180-200 g; Southern Medical University Experimental Animal Center) were acclimated to an SPF facility (temperature 24 ± 2°C, humidity 50%-60%) at Southern Medical University, China. Rats were housed individually with a constant 12 h light/dark cycle (lights on/off at 07:00/19:00) unless otherwise noted. Rats were bred normally for at least 6 days for adaption before the CUMS paradigm. Food and water were available ad libitum. Rats were randomly assigned into 2 groups: control (*n* = 10) and CUMS (*n* = 15).

### 2.2. CUMS Paradigm

**Figure 1:**
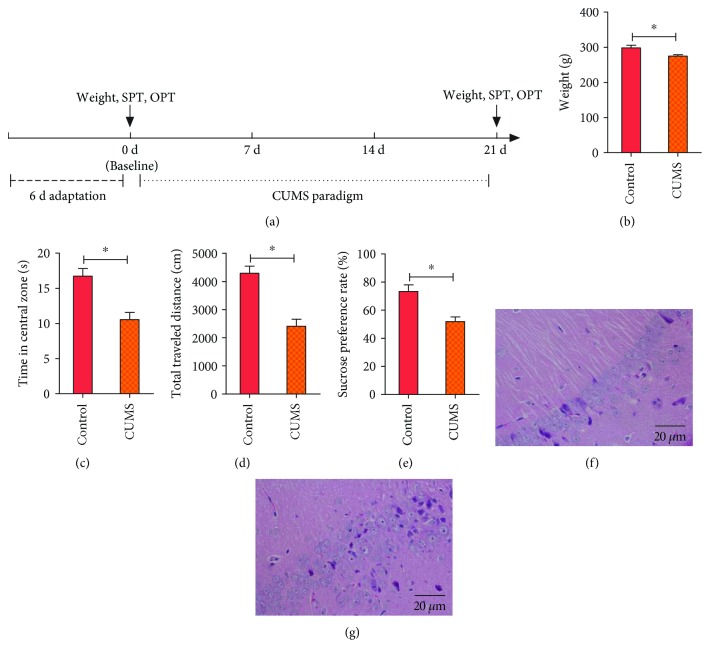
The CUMS paradigm causes depressive behaviors and hippocampal pathology. (a) Timeline of the CUMS paradigm and behavioral assessments. (b–e) CUMS causes depressive behaviors such as weight loss, anxiety, and anhedonia (*n* = 10 − 15 rats/group). (f and g) Hippocampus lesion caused by CUMS shown in CA1 H&E staining (*n* = 6 rats/group). Scale bars: 20 *μ*m. Bar graphs: mean ± SE. ^∗^*P* < 0.05 vs. control. Student's *t*-test.

CUMS rats underwent a 21-day chronic unpredictable mild stress procedure. On each of the 21 consecutive days, rats were exposed to a random stressor (Figure 1(a)). These stressors included water deprivation (24 h), food deprivation (24 h), wet bedding (24 h), light-dark reversal (24 h), stroboscopic lighting (12 h), immobilization (2 h), cold swim (4°C, 5 min), warm swim (45°C, 5 min), level shaking (5 min), and tail clamping (3 min).

### 2.3. Behavioral Experiments

The sucrose preference test (SPT) was used to assess anhedonia. After a 2-day habituation phase, rats were housed singly for 24 h without any food or water. Then, the rats were presented with two identical bottles containing either sucrose solution (1%) or pure water for 1 h. The sucrose preference rate was calculated as the amount of sucrose solution consumed relative to the total fluid consumed.

One day after the SPT, an open-field test (OFT) was performed to evaluate anxiety. The open-field arena (100 × 100 × 40 cm) was equally divided into 25 square areas. The 9 grids in the center were defined as the central region. Rats were individually placed into the arena for 5 min. Distance travelled and time spent in the central zone were analyzed using video cameras with associated software (Smart 2.0).

The behavioral experiments and weighing of the animals were performed before and after the CUMS paradigm (Figure 1(a)).

### 2.4. Hippocampus Tissue Acquisition

After the behavioral experiments, rats were exposed to 25% pentobarbital sodium (50 mg/kg, intraperitoneal injection) and subsequently decapitated. Brains were instantly dissected, and all attached tissues were removed. Hippocampus tissues were separated, rinsed with phosphate-buffered saline (PBS), immediately frozen in liquid nitrogen, and stored at -80°C until analyses (3 rats/group). For hematoxylin and eosin (H&E) staining, cornu ammonis (CA) 1 was separated, fixed in 4% paraformaldehyde, embedded in paraffin, sliced, deparaffinized, and stained for routine H&E staining and histological examination (6 rats/group).

### 2.5. Protein Preparation, iTRAQ Isobaric Labeling, and SCX Separation

Hippocampus tissues were ground into powder in liquid nitrogen using lysis buffer (Roche). Then, the samples were ultrasonically disrupted on ice. Supernatants were collected after centrifugation (10,000*g*, 30 min, 4°C), and protein concentrations were determined using an enhanced BCA (bicinchoninic acid) Protein Assay Kit (P0010; Beyotime Biotechnology Ltd., Beijing, China), according to the manufacturer's instructions. The protein samples (200 *μ*g) were mixed with dl-dithiothreitol, alkylated with iodoacetamide, and then treated with trypsin (protein-trypsin ratio = 50 : 1, 12 h).

Protein peptides (100 *μ*g) from each group were labeled using an iTRAQ Reagent-8plex Multiplex Kit (AB SCIEX, Framingham, MA, USA). The samples were labeled as 113 (control 1), 114 (control 2), 115 (CUMS 1), 116 (CUMS 2), and 117 (CUMS 3). The labeled samples were pooled and further fractionated offline using the ÄKTApurifier 100 (GE Healthcare Life Sciences) with a strong cation exchange column (PolySULFOETHYL A™; PolyLC Inc., Columbia, MD, USA). The retained peptides were eluted with buffer A (10 mM KH_2_PO_4_ in 25% ACN (acetonitrile), pH 3.0) and buffer B (10 mM KH_2_PO_4_ and 500 mM KCl in 25% ACN, pH 3.0) with a flow rate of 0.7 ml/min.

### 2.6. LC-MS/MS Analysis

Eluted fractions were lyophilized using a centrifugal speed vacuum concentrator (CentriVap® Complete Vacuum Concentrator; Labconco, Kansas City, MO, USA) and dissolved in formic acid (5 *μ*l, 0.5%). Equivalent amounts of peptides from each fraction were mixed and then subjected to reversed-phase nanoflow LC-MS/MS analysis using a high-performance liquid chromatography (HPLC) system (EASY-nLC™, Thermo Fisher Scientific) connected to a hybrid quadrupole/time-of-flight mass spectrometer equipped with a nanoelectrospray ion source. The peptides were separated on a C18 analytical reversed-phase column with mixtures of solution A (0.1% formic acid in water) and solution B (0.1% formic acid in ACN). A full MS scan was conducted using a Q Exactive™ mass spectrometer (Thermo Fisher Scientific) with a flow rate of 600 nl/min. Mass spectrometry was then performed using a mass spectrometer (Q Exactive HF, Thermo Fisher Scientific).

### 2.7. Protein Identification and Quantification

Raw MS/MS data were searched against the UniProt database (last modified on April 22, 2017) using Mascot 2.2 and Proteome Discoverer™ 1.4 software (Thermo Fisher Scientific). A peptide false discovery rate (FDR) ≤ 0.01 was used as the identification standard. Protein quantification was based on the total intensity of the assigned peptides. The average of labeled sample mixes was used as a reference and was based on the weighted average of the intensity of the reported ions in each peptide identified. The final protein ratios were normalized to the median average protein content of the 8-plex samples. A 1.2-fold cutoff was set to identify upregulated and downregulated proteins.

### 2.8. Bioinformatics Analysis

The functional enrichment analysis of significantly changed proteins was performed using Gene Ontology (GO) and Kyoto Encyclopedia of Genes and Genomes (KEGG) enrichment analysis with the online software DAVID (https://david.ncifcrf.gov/). Corrected *P* values < 0.1 were considered significantly enriched. Protein-protein interaction (PPI) networks were retrieved from STRING (https://string-db.org/) using Cytoscape software (Version 3.6.1, https://cytoscape.org/). The Markov cluster algorithm (MCL) was then performed to determine topological clusters of the network using Cytoscape software.

### 2.9. Statistical Analysis

All data are expressed as the mean ± SEM. Differences in behavioral results and differentially expressed proteins were evaluated using Student's *t*-test. All statistical analyses were carried out using SPSS software (version 20.0, SPSS Inc., USA). *P* < 0.05 was considered statistically significant.

## 3. Results

### 3.1. Stressors Cause Behavioral and Hippocampal Abnormalities

We used a 21-day chronic unpredictable mild stress (CUMS) paradigm to model human depression in rats. Animals were exposed to chronic unpredictable mild stressors for 21 days (Figure 1(a)). To measure appetite, anxiety, and anhedonia levels, body weight, open-field test (OFT), and sucrose preference test (SPT) were used, respectively.

The CUMS paradigm resulted in a lower weight in CUMS rats than in control rats (Figure 1(b)). CUMS rats also had decreased time spent in the central zone and distance travelled in the OFT, indicating increased anxiety (Figures 1(c) and 1(d)). In the SPT, CUMS rats had a lower sucrose preference rate than control rats, indicating anhedonia (Figure 1(e)).

The hippocampus is one of the important brain regions involved in the biology of depression. Hippocampus tissue was obtained for histology examination. H&E staining showed that control rats had a thick hippocampal pyramidal cell layer as well as densely, closely, and regularly arranged cells in CA1. In contrast, CUMS rats had a thin pyramidal cell layer, widened intercellular spaces, and irregularly and loosely arranged cells. Therefore, tissue damage and cell apoptosis occurred in the hippocampus of CUMS rats (Figures 1(f) and 1(g)).

### 3.2. Changes in the CUMS Hippocampal Proteomic Profile

**Figure 2:**
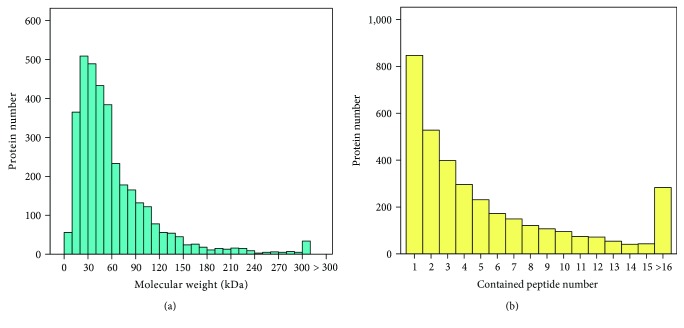
Basic information of protein identification. (a) Histogram of the identified proteins among the different molecular weight classes (in kDa). (b) Histogram of proteins containing different numbers of identified peptides.

To further understand the mechanism of depression, we used iTRAQ to identify differentially expressed hippocampal proteins between groups. Based on the iTRAQ-LC-MS/MS analysis results, a total of 3511 proteins and 18,381 peptides were identified (peptide false discovery rate (FDR) ≤ 0.01). Most of the identified proteins (75.39%) had molecular weights in the range of 10-80 kDa (Figure 2(a)). Approximately 60.84% of the identified proteins had more than 2 peptides (Figure 2(b)).

Fifty-two quantified proteins with *P* < 0.05 and an expression change of higher than 1.50-fold or lower than 0.67-fold between the CUMS and control groups were manually selected (Table 1). Thirty differentially expressed proteins were upregulated, and 22 were downregulated after the CUSM paradigm.

### 3.3. Functional Annotation Enrichment of the Differentially Expressed Proteins

To understand the biological meaning behind the large list of proteins differentially expressed between groups and the underlying mechanism of depression, differently expressed proteins were subjected to enrichment analysis using the DAVID website. The identified enriched biological themes included biological process, molecular function, cellular component, and KEGG pathway. Enrichment analyses were performed on all differentially expressed proteins and then on upregulated and downregulated proteins separately for better understanding.

**Figure 3:**
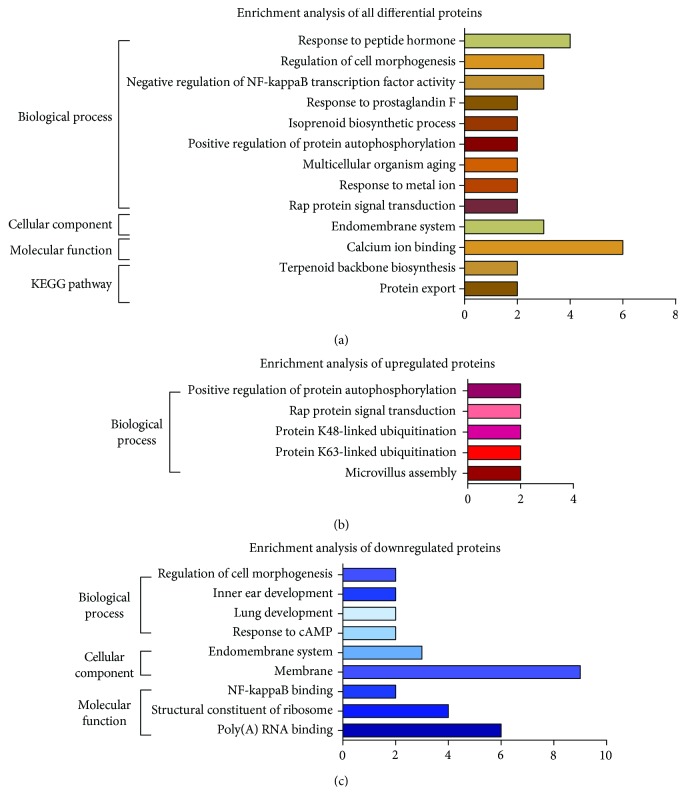
GO ontology annotation and KEGG pathway enrichment analysis of differently expressed proteins. GO annotations and KEGG pathway enrichment analysis of all differentially expressed proteins (a), upregulated proteins (b), and downregulated proteins (c). Scale bar: number of proteins.

For all the differentially expressed proteins, the enrichment analysis results showed that, in terms of biological process, most of the differentially expressed proteins were involved in response to peptide hormone (7.69%, *P* = 0.01), regulation of cell morphogenesis (5.77%, *P* < 0.01), negative regulation of NF-kappaB transcription factor activity (5.77%, *P* = 0.02), response to prostaglandin F (3.85%, *P* = 0.03), isoprenoid biosynthetic process (3.85%, *P* = 0.06), positive regulation of protein autophosphorylation (3.85%, *P* = 0.06), multicellular organism aging (3.85%, *P* = 0.09), response to metal ion (3.85%, *P* = 0.09), and Rap protein signal transduction (3.85%, *P* = 0.09). Regarding molecular function, most of the differentially expressed proteins were annotated as being associated with calcium ion binding (11.54%, *P* = 0.07). In terms of cellular components, most of the differentially expressed proteins were predicted to be in the endomembrane system (5.77%, *P* = 0.06). For KEGG pathways, most of the differentially expressed proteins were involved in terpenoid backbone biosynthesis (3.85%, *P* = 0.08) and protein export (3.85%, *P* = 0.08) (Figure 3(a)).

For upregulated proteins, only biological process enrichment was found, and most of the upregulated proteins were involved in the positive regulation of protein autophosphorylation (6.67%, *P* = 0.03), Rap protein signal transduction (6.67%, *P* = 0.05), protein K48-linked ubiquitination (6.67%, *P* = 0.07), protein K63-linked ubiquitination (6.67%, *P* = 0.07), and microvillus assembly (6.67%, *P* = 0.09) (Figure 3(b)).

For downregulated proteins, enrichment analyses results showed that, in terms of biological process, most of the downregulated proteins were involved in the regulation of cell morphogenesis (9.09%, *P* = 0.04), inner ear development (9.09%, *P* = 0.07), lung development (9.09%, *P* = 0.10), and response to cAMP (9.09%, *P* = 0.10). Regarding molecular function, most of the downregulated proteins were annotated as being associated with NF-kappaB binding (9.09%, *P* = 0.07), the structural constituents of ribosomes (18.18%, *P* = 0.02), and poly(A) RNA binding (27.27%, *P* = 0.10). In terms of the cellular component, most of the downregulated proteins were predicted to be in the endomembrane system (13.64%, *P* = 0.01) and membrane (40.91%, *P* = 0.08) (Figure 3(c)). KEGG pathway enrichment was not found in downregulated proteins.

### 3.4. Protein-Protein Interaction Network of the Differentially Expressed Proteins

**Figure 4:**
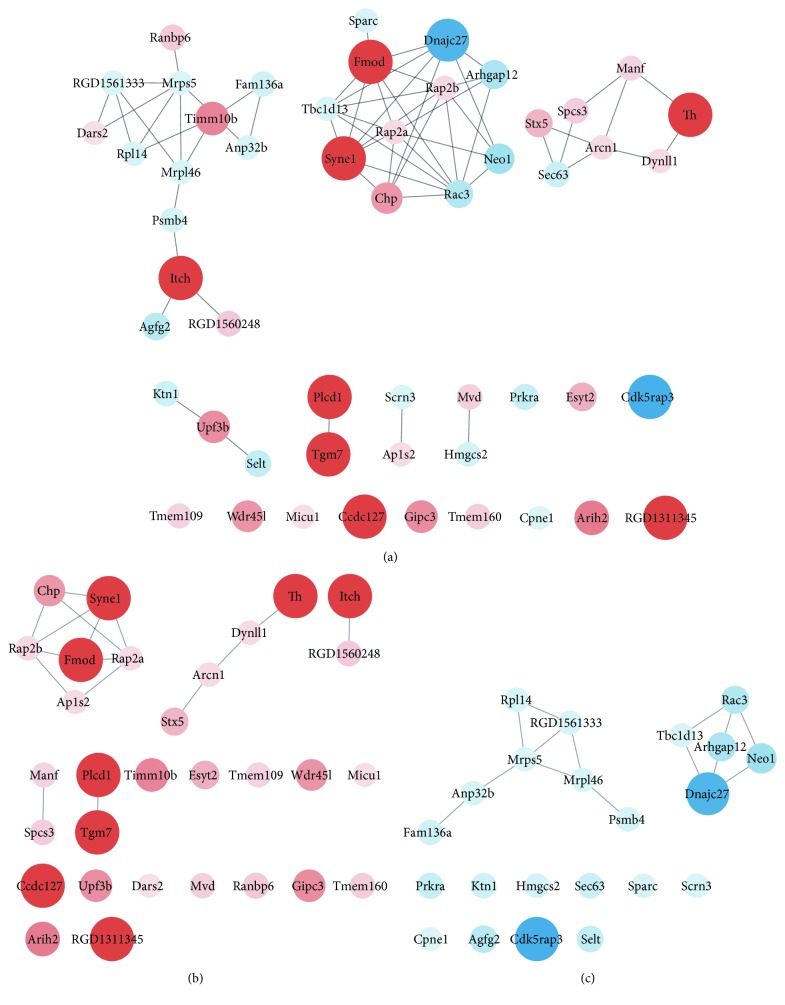
String network with MCL cluster shown. Protein-protein interaction networks with MCL clusters of all differentially expressed proteins (a), upregulated proteins (b), and downregulated proteins (c). Network nodes: proteins (upregulations are represented by red nodes, downregulations are represented by blue nodes, and higher expression changes are represented by larger nodes); edges: associations (stronger associations are represented by darker lines).

First, we retrieved the interaction network of all 52 differentially expressed proteins. MCL was performed to explore the strong connections between groups of nodes. Examining the main connected component of the network, we immediately found that there were 3 clusters of proteins: Psmb4, Mrpl46, Dars2, Ranbp6, Rpl14, Agfg2, Itch, Mrps5, RGD1560248, Timm10b, RGD1561333, and Anp32b; Dnajc27, Chp, Arhgap12, Neo1, Tbc1d13, Rac3, Rap2a, Rap2b, Sparc, Syne1, Fmod, and Fam136a; and Stx5, Manf, Th, Arcn1, Sec63, Spcs3, and Dynll1 (Figure 4(a)).

To further understand the network, we also examined upregulated and downregulated protein networks separately. MCL clustering was also performed. Examining the main connected component of the network of upregulated proteins, we found 2 clusters of proteins, one of which consisted of Rap2b, Rap2a, Chp, Ap1s2, Fmod, and Syne1 (cluster 1). Both Rap2b and Rap2a belong to the family of Ras-related proteins, also known as Rap GTP-binding protein, one of the subfamilies of the Ras superfamily (Table 2). Members of this superfamily appear to regulate a diverse array of cellular events, including cell growth control, cytoskeletal reorganization, and protein kinase activation.

The other cluster consisted of Th, Stx5, Dynll1, and Arcn1 (cluster 2) (Figure 4(b)). These proteins are mainly involved in vesicle structure and trafficking. For instance, Stx5 is a member of the syntaxin or t-SNARE (target-SNAP receptor) family, which plays a crucial role in synaptic vesicle docking. Notably, Th is a rate-limiting enzyme in the synthesis of catecholamines, which is the process necessary for the formation of the dopamine (DA) precursor levodopa (l-DOPA). Hence, Th plays a key role in the biosynthesis of dopamine (Table 2).

As for the network of downregulated proteins, one cluster of downregulated proteins contained Mrps5, Psmb4, Mrpl46, RGD1561333, Anp32b, Fam136a, and Rpl14 (cluster 3). These proteins are mostly ribosome translation related. For example, Mrps5, Mrpl46, and Rpl14 are all ribosomal subunit proteins, and RGD1561333 and Anp32b are both involved in translation (Table 3).

There was also a cluster of proteins, Rac3, Tbc1d13, Neo1, Arhgap12, and Dnajc27, that was predominantly downregulated (Figure 4(c)), and which was mainly relevant to the Ras superfamily of small GTP-binding proteins and subsequent signaling pathways. For example, Rac3 and Dnajc27 belong to the Rho and Rab protein families, respectively, which are subfamilies belonging to the Ras superfamily. Tbc1d13 binds to Rab GTPase (Table 3).

## 4. Discussion

After exposure to stressors for 21 days, the CUMS rats exhibited less time spent in the central zone, less distance travelled, and lower sucrose preference in the OFT and SPT, as well as decreased weight, indicating elevated anhedonia and anxiety levels. Hippocampus lesions were also observed. These results suggest that the depression model was successfully established. To understand the hippocampal proteomic changes underlying the mechanism of depression, we used LC-MS/MS analysis and bioinformatics analysis to identify the significantly changed proteins between the CUMS and control groups. We found GO enrichment in the GO term “Rap protein (a subfamily of Ras superfamily) signal transduction” among all differently expressed proteins and in the “structural constituent of ribosome” among downregulated proteins (Figure 3). Similarly, in the MCL cluttering analyses, some identified clusters are involved in ribosomal translation and are relevant to the Ras superfamily (Figure 4). Together, these findings suggest that hippocampal ribosome lesions and Ras protein changes underlie the mechanism of depression.

### 4.1. Ribosome and Depression

Ribosomes serve as the workplace of RNA translation, which makes them vital organelles for protein synthesis [22]. In the neural system, ribosomes are known to contribute to neuron development. Moreover, rapid, local activation of protein synthesis in ribosomes is required for synaptic plasticity [23]. Ribosomes not only exist in the soma of neurons but also play an important role in axons and synapses. RNA is transferred to its postsynaptic destination and subsequently translated in the postsynaptic ribosome [24]. A recent study also demonstrated that presynaptic protein synthesis in the ribosome is essential for the long-term plasticity of neurotransmitter gamma-aminobutyric acid (GABA) release [25].

In our study, the expression of ribosome proteins was significantly decreased in the hippocampus of depressed (CUMS) rats, especially the expression of ribosomal subunit proteins Mrps5, Mrpl46, and Rpl14 (Figure 4(a)). Similar studies have also revealed ribosome lesions in depression patients and animal models [26–28]. Interestingly, both Mrps5 and Mrpl46 belong to the family of mitochondrial ribosomal proteins (MRPs).

Research has found that MRPs are evolutionarily conserved proteins that serve as metabolic and longevity regulators. MRPs play a crucial role in activating the mitochondrial unfolded protein response (UPR^mt^) and therefore maintaining the balance of mitochondrial-nuclear proteins and extending lifespan [29]. Lifespan enhancers such as rapamycin and resveratrol also share this mechanism [30]. UPR^mt^ activation has been observed in a mouse model of depression caused by chronic restraint [31]. These studies and ours provide a new strategy in depression intervention to use rapamycin and resveratrol as supplements to alleviate depression by changing mitochondrial translation.

### 4.2. Ras Superfamily in Depression

The Ras superfamily is an evolutionarily conserved protein superfamily of small GTPases, including several subfamilies, such as Ras, Rho, Ran, Rab, and Arf GTPases, among which the Ras family itself is further divided into Ras, Ral, Rap, Rheb, Rad, and the recently included Rit and *Miro* [32]. Generally, these proteins are responsible for cell proliferation and survival [33]. Until now, the mechanism of Ras family proteins has primarily been discussed in terms of their role in tumorigenesis. However, recent studies have shown that the Ras superfamily is involved in psychiatric disorders. Ras gene mutations are found in patients suffering from psychiatric and neurodevelopmental disorders [34].

Ras proteins activate and stimulate multiple downstream effector pathways by direct interactions, such as the Raf/Mitogen-activated protein kinase kinase (MEK)/extracellular regulated protein kinase (ERK) cascade and the phosphoinositide 3-kinase (PI3K) signaling cascades [35]. These pathways mediate the control of various physiological processes. Taking PI3K signaling cascades as an example, the pathway has been found to be a necessary component in LTP [36]. Studies have also shown that these signaling cascades serve as key biochemical cascades in *α*-amino-3-hydroxy-5-methyl-4-isoxazolepropionic acid receptor (AMPAR) trafficking during synaptic plasticity in neurons and altered behavior [37].

Altered hippocampal synaptic plasticity is considered one of the underlying mechanisms of depression. In our research, the expression of proteins in the Ras superfamily changed significantly. The MCL clustering results showed a mixed change in these proteins, meaning some of the Ras proteins were upregulated and some downregulated. Upregulated proteins such as Rap2b and Rap2a belong to the Rap family (Figure 4(a)).

Notably, Ras and Rap proteins of the Ras subfamily function antagonistically [38]. In neurons, Ras plays a crucial role in synapse enforcement and LTP by promoting postsynaptic insertion of AMPAR. Rap weakens synapses and induces long-term depression (LTD) by increasing AMPAR internalization [39]. Our results show that typical Rap proteins, Rap2b and Rap2a, were upregulated in the CUMS hippocampus (Table 2), which indicates synaptic weakening and synaptic plasticity disturbances in depression.

On the other hand, of the downregulated proteins identified, Rac3 belongs to the Rho family and Dnajc27 belongs to the Rab family (Table 3). Specifically, Rho proteins are responsible for the morphogenesis of dendritic spines [40] and Rab for that of synaptic vesicles [41], which are both vital biological processes underlying synaptic plasticity. Therefore, we consider that Ras proteins are involved in hippocampal pathology changes by affecting hippocampal synaptic plasticity.

However, we did not conduct experiments examining the UPR^mt^ or synaptic plasticity of hippocampal neurons in this study. Whether these proteins are responsible for the UPR^mt^ and disrupted synaptic plasticity in the hippocampus is still unknown. Further research may be needed to draw a conclusion. Another possible limitation of the study is that we did not focus on a specific subfield of the hippocampus, such as the dentate gyrus (DG), CA1, CA2, CA3, or CA4. Because most of the identified proteins in this research are synapse-related, we would like to focus on the CA1 and DG in future studies. Indeed, synaptic plasticity in the CA1 is rather vulnerable in diseases, and adult neurogenesis still exists in the DG, which makes DG a subfield of high synaptic plasticity [42, 43].

## Figures and Tables

**Table 1:** Differentially expressed proteins between the CUMS and control groups.

Uniprot accession	Protein name	Gene	Fold change (CUMS/control)	*P*
F1LZP5	Protein Tgm7	Tgm7l1	Undetected in control group	/
G3V7Q3	Coiled-coil domain-containing protein 127	Ccdc127	Undetected in control group	/
Q5YB86	Itch E3 ubiquitin ligase	Itch	Undetected in control group	/
B4F797	RGD1311345 protein (fragment)	RGD1311345	Undetected in control group	/
G3V6E7	Fibromodulin	Fmod	Undetected in control group	/
Q8VHJ9	Nesprin-1	Syne1	Undetected in control group	/
P04177	Tyrosine 3-monooxygenase	Th	Undetected in control group	/
P10688	1-Phosphatidylinositol 4,5-bisphosphate phosphodiesterase delta-1	Plcd1	Undetected in control group	/
D3ZJB8	Ariadne homolog 2 (*Drosophila*) (predicted), isoform CRA_a	Arih2	2.15	0.04
Q9R1B1	Mitochondrial import inner membrane translocase subunit Tim10 B	Timm10b	2.05	0.01
D3ZEK6	Protein Gipc3	Gipc3	1.97	0.04
D3ZBE8	Protein Upf3b	Upf3b	1.96	0.01
Q2MCP5	Protein Wdr45b	Wdr45b	1.88	0.01
P61023	Calcineurin B homologous protein 1	Chp1	1.86	0.001
D3ZJ32	Protein Esyt2	Esyt2	1.61	0.02
Q08851	Syntaxin-5	Stx5	1.56	0.01
D4A634	Protein Ranbp6	Ranbp6	1.37	0.03
A0A0G2K132	Protein Fmnl2	Fmnl2	1.37	0.01
D3ZZU4	Protein Tmem160	Tmem160	1.35	0.05
Q568Z4	Signal peptidase complex subunit 3	Spcs3	1.34	0.02
Q99PJ4	Diphosphomevalonate decarboxylase (fragment)	Mvd	1.33	0.02
Q6AYQ4	Transmembrane protein 109	Tmem109	1.30	0.03
P0C5H9	Mesencephalic astrocyte-derived neurotrophic factor	Manf	1.29	0.02
P61227	Ras-related protein Rap-2b	Rap2b	1.25	0.03
A0A0G2JTW1	Protein Rap2a	Rap2a	1.25	0.04
Q66H80	Coatomer subunit delta	Arcn1	1.25	0.04
P63170	Dynein light chain 1, cytoplasmic	Dynll1	1.24	0.02
B2GV08	Adaptor-related protein complex 1, sigma 2 subunit (predicted), isoform CRA_a	Ap1s2	1.23	0.02
F1LNV5	Calcium uptake protein 1, mitochondrial	Micu1	1.22	0.02
Q3KRD0	Aspartate-tRNA ligase, mitochondrial	Dars2	1.20	0.03
Q642E3	CDK5 regulatory subunit associated protein 3, isoform CRA_b	Cdk5rap3	Undetected in CUMS group	/
D4A1R8	Copine-1	Cpne1	0.83	0.01
D3ZYT2	Mitochondrial ribosomal protein S5 (predicted)	Mrps5	0.82	0.01
D4AEG7	Protein Tbc1d13	Tbc1d13	0.81	0.02
A0A0G2K189	Protein Scrn3	Scrn3	0.81	0.01
Q5RK00	39S ribosomal protein L46, mitochondrial	Mrpl46	0.81	0.01
P16975	SPARC	Sparc	0.81	0.02
D4A6W6	Protein RGD1561333	RGD1561333	0.80	0.03
F1LSW7	60S ribosomal protein L14	Rpl14	0.80	0.01
F1LP34	Acidic leucine-rich nuclear phosphoprotein 32 family member B	Anp32b	0.80	0.03
G3V8U9	Proteasome subunit beta type	Psmb4	0.79	0.05
P22791	Hydroxymethylglutaryl-CoA synthase, mitochondrial	Hmgcs2	0.79	0.02
B0BN94	Protein FAM136A	Fam136a	0.76	0.03
D4A4Z9	Protein Ktn1	Ktn1	0.75	0.02
D4A2Z6	Protein Sec63	Sec63	0.74	0.03
G3V7J2	Interferon-inducible double-stranded RNA-dependent protein kinase activator A	Prkra	0.72	0.02
Q5PQZ8	Selenoprotein T	Selt	0.70	0.02
A0A0G2K7G2	Protein Agfg2	Agfg2	0.66	0.03
M0R5T4	Protein Rac3	Rac3	0.64	0.02
A0A0G2K1Z2	Protein Arhgap12	Arhgap12	0.61	0.05
F1M0Z6	Neogenin	Neo1	0.57	0.04
Q6IML7	DnaJ homolog subfamily C member 27	Dnajc27	0.35	0.01

**Table 2:** MCL clusters of upregulated proteins.

Cluster	Gene/displayed name	Biological process	Molecular function	Cellular component
1	Syne1	Golgi organization, brain development, response to light stimulus, establishment of nucleus localization, muscle cell differentiation, positive regulation of receptor-mediated endocytosis, regulation of dendrite morphogenesis, cytoskeletal anchoring at nuclear membrane, nuclear matrix anchoring at nuclear membrane	Actin binding, receptor binding, structural molecule activity, protein binding, lamin binding, enzyme binding, identical protein binding, protein homodimerization activity, poly(A) RNA binding, actin filament binding	Nucleus, nuclear envelope, nucleoplasm, cytoplasm, Golgi apparatus, spindle, integral component of membrane, sarcomere, midbody, nuclear membrane, LINC complex, dendritic spine, postsynaptic membrane, perinuclear region of cytoplasm
	Rap2b	Microvillus assembly, platelet activation, negative regulation of cell migration, positive regulation of protein autophosphorylation, Rap protein signal transduction, regulation of protein tyrosine kinase activity, platelet aggregation, establishment of endothelial intestinal barrier	GTP binding, GDP binding, protein domain-specific binding	Cytosol, plasma membrane, bicellular tight junction, membrane, cell-cell contact zone, membrane raft, recycling endosome, recycling endosome membrane, extracellular exosome
	Rap2a	Positive regulation of protein phosphorylation, small GTPase-mediated signal transduction, microvillus assembly, negative regulation of cell migration, actin cytoskeleton reorganization, positive regulation of protein autophosphorylation, Rap protein signal transduction, cellular protein localization, cellular response to drug, establishment of protein localization, establishment of epithelial cell apical/basal polarity, regulation of JNK cascade, regulation of dendrite morphogenesis, protein localization to plasma membrane	GTPase activity, GTP binding	Intracellular, cytosol, plasma membrane, membrane, recycling endosome, recycling endosome membrane, extracellular exosome
	Chp	Microtubule bundle formation, negative regulation of protein phosphorylation, negative regulation of protein kinase activity, protein export from nucleus, negative regulation of phosphatase activity, calcium ion-regulated exocytosis, calcium-mediated signaling, membrane docking, cytoplasmic microtubule organization, negative regulation of protein ubiquitination, negative regulation of protein autophosphorylation, negative regulation of NF-kappaB transcription factor activity, positive regulation of sodium : proton antiporter activity, negative regulation of protein import into nucleus, transcytosis, protein stabilization, positive regulation of protein transport, protein oligomerization, regulation of intracellular pH, positive regulation of protein glycosylation, membrane organization, membrane fusion, negative regulation of calcineurin-NFAT signaling cascade, cellular response to acidic pH, positive regulation of protein targeting to membrane, regulation of neuron death	Protein kinase inhibitor activity, transporter activity, calcium ion binding, protein binding, microtubule binding, kinase binding, calcium-dependent protein binding	Golgi membrane, nucleus, cytoplasm, endoplasmic reticulum, endoplasmic reticulum-Golgi intermediate compartment, cytosol, plasma membrane, focal adhesion, microtubule cytoskeleton, transport vesicle, extracellular exosome
	Ap1s2	Intracellular protein transport, visual learning, vesicle-mediated transport, synaptic vesicle recycling, fat cell differentiation, neuromuscular process controlling balance, adipose tissue development	Protein transporter activity	Golgi apparatus, membrane coat, intracellular membrane-bounded organelle

2	Dynll1	Transcription, DNA-templated, regulation of transcription, DNA-templated, nitric oxide biosynthetic process, transport, apoptotic process, microtubule-based process, substantia nigra development, intraciliary retrograde transport, neurotransmitter metabolic process, negative regulation of phosphorylation, negative regulation of catalytic activity, motile cilium assembly, cilium morphogenesis, positive regulation of nonmotile primary cilium assembly	Motor activity, enzyme inhibitor activity, protein binding, protein C-terminus binding, enzyme binding, protein domain-specific binding, nitric oxide synthase regulator activity, protein homodimerization activity, dynein intermediate chain binding	Kinetochore, nucleus, cytoplasm, mitochondrion, centrosome, cytosol, cytoskeleton, cytoplasmic dynein complex, microtubule, cilium, COP9 signalosome, membrane, extracellular exosome, mitotic spindle
	Arcn1	Retrograde vesicle-mediated transport, Golgi to ER, adult locomotor behavior, protein transport, cerebellar Purkinje cell layer maturation, pigmentation, Golgi vesicle transport	Poly(A) RNA binding	Golgi membrane, cytoplasm, endoplasmic reticulum, Golgi apparatus, membrane, COPI vesicle coat, COPI-coated vesicle, intracellular membrane-bounded organelle
	Th	Response to hypoxia, synaptic transmission, dopaminergic, response to amphetamine, dopamine biosynthetic process from tyrosine, fatty acid metabolic process, sphingolipid metabolic process, heart development, visual perception, sensory perception of sound, learning, memory, mating behavior, locomotor behavior, regulation of heart contraction, response to water deprivation, response to light stimulus, response to herbicide, response to salt stress, organ morphogenesis, response to metal ion, response to zinc ion, multicellular organism aging, response to organic cyclic compound, response to activity, aminergic neurotransmitter loading into synaptic vesicle, glycoside metabolic process, response to insecticide, phthalate metabolic process, cerebral cortex development, response to nutrient levels, response to estradiol, response to lipopolysaccharide, isoquinoline alkaloid metabolic process, response to nicotine, social behavior, cellular response to drug, response to isolation stress, response to immobilization stress, neurotransmitter biosynthetic process, terpene metabolic process, dopamine biosynthetic process, epinephrine biosynthetic process, norepinephrine biosynthetic process, catecholamine biosynthetic process, eye photoreceptor cell development, response to drug, circadian sleep/wake cycle, eating behavior, response to peptide hormone, response to ethanol, response to ether, response to pyrethroid, response to steroid hormone, embryonic camera-type eye morphogenesis, cognition, protein homotetramerization, response to corticosterone, response to electrical stimulus, phytoalexin metabolic process, oxidation-reduction process, response to growth factor, cellular response to manganese ion, cellular response to alkaloid, cellular response to nicotine, cellular response to glucose stimulus, cellular response to growth factor stimulus	Monooxygenase activity, tyrosine 3-monooxygenase activity, protein binding, ferrous iron binding, ferric iron binding, amino acid binding, oxygen binding, enzyme binding, protein domain-specific binding, tetrahydrobiopterin binding, dopamine binding	Nucleus, cytoplasm, mitochondrion, smooth endoplasmic reticulum, cytosol, synaptic vesicle, cytoplasmic side of plasma membrane, axon, dendrite, cytoplasmic vesicle membrane, cytoplasmic vesicle, melanosome membrane, neuron projection, neuronal cell body, terminal bouton, perikaryon
	Stx5	Intracellular protein transport, ER to Golgi vesicle-mediated transport, early endosome to Golgi transport, retrograde transport, endosome to Golgi, positive regulation of protein catabolic process, vesicle docking, vesicle fusion with Golgi apparatus, Golgi disassembly, cell-cell adhesion, regulation of Golgi organization	SNARE binding, SNAP receptor activity, protein binding, protein N-terminus binding, cadherin binding involved in cell-cell adhesion	Golgi membrane, nucleoplasm, endoplasmic reticulum, Golgi apparatus, cytosol, cell-cell adherens junction, integral component of membrane, SNARE complex, vesicle, endoplasmic reticulum-Golgi intermediate compartment membrane

**Table 3:** MCL clusters of downregulated proteins.

Cluster	Gene/displayed name	Biological process	Molecular function	Cellular component
3	Mrps5	Translation	Structural constituent of ribosome, poly(A) RNA binding	Mitochondrion, mitochondrial small ribosomal subunit, cytosolic small ribosomal subunit
	Psmb4	Negative regulation of inflammatory response to antigenic stimulus, proteolysis involved in cellular protein catabolic process	Lipopolysaccharide binding, threonine-type endopeptidase activity	Proteasome complex, nucleus, cytoplasm, proteasome core complex, extracellular exosome
	Mrpl46	/	Structural constituent of ribosome, hydrolase activity	Nucleoplasm, mitochondrion, mitochondrial large ribosomal subunit, cell junction
	RGD1561333	Cytoplasmic translation	RNA binding, structural constituent of ribosome, poly(A) RNA binding	Nucleolus, focal adhesion, membrane, cytosolic large ribosomal subunit
	Anp32b	Vasculature development, nucleosome assembly, nucleocytoplasmic transport, activation of cysteine-type endopeptidase activity involved in apoptotic process, positive regulation of cell proliferation, ventricular system development, negative regulation of apoptotic process, regulation of cysteine-type endopeptidase activity involved in apoptotic process, histone exchange, negative regulation of cell differentiation, positive regulation of protein export from nucleus, inner ear development, palate development, positive regulation of G1/S transition of mitotic cell cycle	Protein binding, histone binding, RNA polymerase binding	Nucleus, nucleolus, cytoplasm, extracellular exosome
	Fam136a	/	/	Cytoplasm, mitochondrion
	Rpl14	rRNA processing, translation, ribosomal large subunit biogenesis	Structural constituent of ribosome, poly(A) RNA binding	Cytoplasm, ribosome, membrane, cytosolic large ribosomal subunit, extracellular exosome

4	Tbc1d13	Intracellular protein transport, regulation of vesicle fusion, activation of GTPase activity, regulation of cilium assembly	GTPase activator activity, Rab GTPase binding	Intracellular, endomembrane system
	Neo1	Neuron migration, regulation of transcription, DNA templated, cell adhesion, axon guidance, myoblast fusion, positive regulation of BMP signaling pathway, regulation of axon regeneration, negative regulation of axon regeneration, negative regulation of protein secretion, iron ion homeostasis, negative regulation of neuron death, regulation of neuron migration	Receptor activity, signaling receptor activity, coreceptor binding, cadherin binding, BMP receptor binding	Nucleoplasm, Golgi apparatus, plasma membrane, integral component of plasma membrane, cell surface, membrane, neuronal cell body, intracellular vesicle, plasma membrane protein complex
	Arhgap12	Morphogenesis of an epithelial sheet, signal transduction	/	/
	Dnajc27	Small GTPase-mediated signal transduction, positive regulation of MAPK cascade, positive regulation of ERK1 and ERK2 cascades, regulation of MAPK export from nucleus	GTPase activity, GTP binding	Nucleus, mitochondrion

## Data Availability

The data used to support the findings of this study are available from the corresponding authors upon request.
